# Effect of *ADORA2A* Gene Polymorphism and Acute Caffeine Supplementation on Hormonal Response to Resistance Exercise: A Double-Blind, Crossover, Placebo-Controlled Study

**DOI:** 10.3390/nu16121803

**Published:** 2024-06-08

**Authors:** Mohammad Rahman Rahimi, Ekaterina A. Semenova, George John, Fateme Fallah, Andrey K. Larin, Edward V. Generozov, Ildus I. Ahmetov

**Affiliations:** 1 Department of Exercise Physiology, University of Kurdistan, Sanandaj 66177-15175, Iran; 2 Department of Molecular Biology and Genetics, Lopukhin Federal Research and Clinical Center of Physical-Chemical Medicine of Federal Medical Biological Agency, 119435 Moscow, Russia; 3 Research Institute of Physical Culture and Sport, Volga Region State University of Physical Culture, Sport and Tourism, 420138 Kazan, Russia; 4 Transform Specialist Medical Centre, Dubai 119190, United Arab Emirates; 5 Laboratory of Genetics of Aging and Longevity, Kazan State Medical University, 420012 Kazan, Russia; 6 Sports Genetics Laboratory, St. Petersburg Research Institute of Physical Culture, 191040 St. Petersburg, Russia; 7 Research Institute for Sport and Exercise Sciences, Liverpool John Moores University, Liverpool L3 5AF, UK

**Keywords:** nutrigenetics, nutrigenomics, DNA, polymorphism, anabolic hormones, caffeine, individual responses, sports nutrition, exercise

## Abstract

Previous studies have reported that TT genotype carriers of the adenosine A2a receptor (*ADORA2A*) gene rs5751876 polymorphism have better ergogenic and anti-inflammatory responses to caffeine intake compared to C allele carriers. The aim of the present study was twofold: (1) to investigate the association of the *ADORA2A* rs5751876 polymorphism with acute caffeine supplementation on hormonal (growth hormone and testosterone) response to resistance exercise (RE); (2) to examine the relationship between the rs5751876 polymorphism and the resting levels of growth hormone and testosterone in athletes who are light caffeine consumers. A double-blind, crossover, placebo-controlled study involving 30 resistance-trained men (age 21.7 ± 4.1) was conducted to assess the impact of caffeine supplementation on serum growth hormone (GH) and testosterone (TS) levels before, immediately after, and 15 min post-RE. One hour before engaging in resistance exercise, subjects were randomly administered 6 mg of caffeine per kg of body mass or a placebo (maltodextrin). After a 7-day washout period, the same protocol was repeated. Resting testosterone and growth hormone levels were examined in the sera of 94 elite athletes (31 females, age 21.4 ± 2.8; 63 males, age 22.9 ± 3.8). Caffeine consumption led to significantly greater increases in GH and TS in men with the TT genotype compared to C allele carriers. Furthermore, in the group of athletes, carriers of the TT genotype had significantly higher testosterone (*p* = 0.0125) and growth hormone (*p* = 0.0365) levels compared to C allele carriers. In conclusion, the *ADORA2A* gene rs5751876 polymorphism may modify the effect of caffeine intake on the hormonal response to exercise.

## 1. Introduction

Caffeine (CAF), scientifically known as 1,3,7-trimethylxanthine, is a stimulating substance found in various popular consumables, including coffee, tea, chocolate, and energy drinks [1]. Since 2004, caffeine has been widely adopted as a legal performance enhancer by both competitive and recreational athletes [2]. Upon ingestion, caffeine is rapidly absorbed through the small intestine, reaching peak concentration on average within 30 min [3]. In humans, caffeine’s half-life—the time it takes for half of the substance to be eliminated from the body—is approximately 3 to 5 h [4]. Caffeine is metabolized swiftly in the liver into three primary metabolites: paraxanthine (84%), theophylline (4%), and theobromine (12%), all of which linger in the bloodstream longer than caffeine itself [3].

Paraxanthine, similar to caffeine, acts as an inhibitor of phosphodiesterase, an enzyme critical in the breakdown of cyclic adenosine monophosphate (cAMP) [1]. When phosphodiesterase is inhibited, there is an increase in cAMP levels, which in turn stimulates the breakdown of fats (lipolysis) and the conversion of glycogen to glucose (glycogenolysis). Furthermore, paraxanthine enhances the secretion of catecholamines, influencing the movement of calcium ions in muscle tissue. These effects—the elevation of cAMP and the increased permeability to calcium ions—contribute to enhanced muscle contractility [5].

Research has shown that resistance exercise (RE) prompts acute fluctuations in hormones (i.e., GH and TS) and leads to long-term adaptations [6], such as hormonal changes that are critical in the muscle rebuilding process. These hormonal responses play an essential role in the synthesis and degradation of myofibrillar proteins [7]. Anabolic hormones like testosterone (TS), growth hormone (GH), insulin, and insulin-like growth factor-I are regulators of protein synthesis. In contrast, cortisol is a catabolic hormone that promotes muscle breakdown and hinders protein synthesis [8,9].

Adhering to the principle of overload during resistance training is known to elevate the levels of anabolic hormones such as testosterone and insulin-like growth factor, as well as enhance neuromuscular function [10,11,12]. The surge in anabolic hormones is typically influenced by various factors of resistance training, including the intensity of the load, the training volume (number of sets and repetitions per set), rest duration between sets, and the total muscle groups engaged [11,13,14,15,16,17,18,19,20].

The performance-enhancing effects of CAF consumption on resistance exercise outcomes have been debated. A meta-analysis indicated a noticeable improvement in overall strength (effect size [ES] = 0.20) and power (ES = 0.17) following caffeine supplementation (4.7 mg/kg [range 0.9–6]), yet a more detailed analysis revealed that this improvement was significant only for upper body strength (ES = 0.21), with no noteworthy impact on lower body strength (ES = 0.15) [21]. As for caffeine’s influence on growth hormone and testosterone responses post-resistance exercise, studies have reported mixed results. Some researchers have observed an increase in concentrations of GH and TS [22,23,24], while other studies have found no change or even a reduction in their levels [25,26] following CAF supplementation in conjunction with resistance training activities.

According to the mentioned findings, it seems that the effect of CAF supplementation and its influence on hormonal reactions vary considerably among individuals. This variation is thought to be due to genetic disparities, with particular attention given to genetic variants (i.e., DNA polymorphisms) involved in caffeine processing, such as the *CYP1A2* gene [27], and the *ADORA2A* gene as cited by [28] and Loy et al. [29]. 

The *ADORA2A* gene encodes the A2AR receptor, which plays an important role in many biological functions, including sensitivity to caffeine. A specific genetic variant within this gene, the single nucleotide polymorphism rs5751876 (also known as 1976 T > C), distinguishes individuals as either “fast” or “slow” caffeine responders based on the carriage of TT or CC/CT genotypes, respectively. People with the TT genotype tend to consume more caffeine, and may exhibit enhanced performance when taking caffeine supplements compared to those with the C allele, as noted previously [30,31,32].

However, the specific influence of the *ADORA2A* gene’s rs5751876 variation on the response of anabolic hormones like growth hormone (GH) and testosterone (TS) following resistance exercise remains uncertain. It is also unknown whether this polymorphism affects the hormonal levels of athletes who regularly and moderately consume caffeine. The aim of the present study, therefore, was twofold: (1) to investigate the association of the *ADORA2A* rs5751876 polymorphism with acute caffeine supplementation on hormonal (growth hormone and testosterone) response to resistance exercise (RE); and (2) to examine the relationship between the rs5751876 polymorphism and the resting levels of growth hormone and testosterone in athletes who are light caffeine consumers.

## 2. Materials and Methods

### 2.1. Ethics Statement

The study was approved by the Ethics Committee in Biomedical Research at the University of Kurdistan (IR.UOK.REC.1397.027) and the Ethics Committee of the Federal Research and Clinical Center of Physical-Chemical Medicine of the Federal Medical and Biological Agency of Russia (approval number 2017/04). Written informed consent was obtained from each participant. The studies complied with the guidelines set out in the Declaration of Helsinki and ethical standards in sport and exercise science research.

### 2.2. Participants

#### 2.2.1. Resistance Exercise Study

In this investigation, 30 men (age 21.7 ± 4.1 years; height 179.3 ± 5.1 cm; weight 77.3 ± 14.7 kg; BMI 24.1 ± 4.1 kg/m^2^; basal metabolic rate 2080 ± 229 kcal, fat mass 11.6 ± 6.5 kg) with a background of at least one year in resistance training were randomly selected for participation. The participants engaged in a minimum of three weekly resistance training sessions, and were categorized as light caffeine (CAF) consumers based on criteria by Haskell et al. [33] (i.e., consumed ~50 mg caffeine/day).

The exclusion criteria included smoking; recent use of CAF-containing drugs; high daily CAF intake (≥70 mg); CAF supplement consumption (energy drinks/pre workouts/caffeine pills and bars); and the use of anabolic hormone-boosting supplements. The study employed a randomized, double-blind, crossover, and placebo-controlled design to assess the influence of the *ADORA2A* gene rs5751876 polymorphism on testosterone (TS), growth hormone (GH) responses, and exercise performance. Participants were assigned to either CAF (6 mg/kg body weight provided by Sigma-Aldrich, Darmstadt, Germany) or placebo (PL; 6 mg/kg body weight maltodextrin) groups, using an online randomization tool (https://www.randomizer.org/, accessed on 25 April 2024). The registration number is IR.UOK.REC.1397.027.

#### 2.2.2. Cross-Sectional Study

Resting testosterone and growth hormone levels were examined in the sera of 94 Russian athletes (31 females, age 21.4 ± 2.8 years, height 170.8 ± 8.6 cm, weight 63.5 ± 8.6 kg, BMI 21.6 ± 2.5 kg/m^2^; 63 males, age 22.9 ± 3.8 years, height 185.9 ± 10.8 cm, weight 82.3 ± 11.3 kg, BMI 23.9 ± 1.9 kg/m^2^). This group was composed of elite athletes (national team members participating in international competitions), including rowers (*n* = 15), badminton players (*n* = 7), baseball players (*n* = 7), biathletes (*n* = 6), wrestlers (*n* = 5), cyclists (*n* = 5), volleyball players (*n* = 4), kayakers (*n* = 7), speed skaters (*n* = 5), cross-country skiers (*n* = 12), swimmers (*n* = 17), and weightlifters (*n* = 4). The athletes were from the same team and trained under the supervision of the same coach (within each sporting discipline). All of the athletes were light caffeine consumers (~50 mg caffeine/day).

### 2.3. Resistance Exercise Study Design

**Figure 1:**
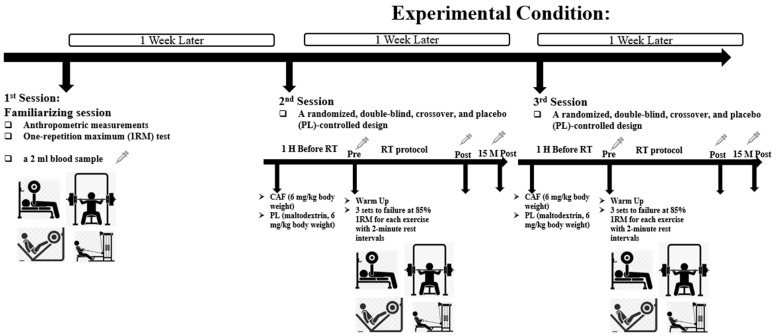
Study design.

Three experimental trials were conducted, spaced one week apart. The initial session involved familiarizing participants with the study protocol and determining their maximum strength via the one-repetition maximum (1RM) method for bench press (BP), leg press (LP), seated cable row (SCR), and shoulder press (SP) [34]. In the subsequent sessions, participants ingested a gelatin capsule containing either CAF (6 mg/kg body weight) or PL (maltodextrin, 6 mg/kg body weight) with 250 mL of water one hour before exercise, to coincide with peak blood CAF levels (Figure 1). Trials were conducted in the afternoons to control for circadian variance.

The warm-up protocol included 5 min of jogging (low intensity), static stretching (including upper and lower body), and joint range of motion exercises (chest/shoulders/back), followed by a set of five repetitions (50% of 1RM) for BP, LP, SCR, and SP. The resistance training regimen consisted of three sets to failure at 85% 1RM for each exercise with 2-minute rest intervals, under both CAF and PL conditions. Performance was monitored and verbally encouraged throughout by the same supervisor, and the number of repetitions completed in each set was recorded.

### 2.4. Genotyping

#### 2.4.1. Resistance Exercise Study

To ascertain the genetic profile of each study participant, a 2 mL blood sample was collected into an EDTA-containing tube from the brachial vein during the initial familiarization session. Once all of the samples were collected, they were transported to a genetic analysis facility. The genomic DNA was extracted utilizing the TIANamp Genomic DNA Kit (Tiangen Biotech, Beijing, China) according to the provided instructions (Cat. No. DP304). Given the significance of DNA quality for molecular studies, each DNA sample underwent qualitative assessment. For this qualitative analysis, the extracted DNA was evaluated on a 1% agarose gel. A volume of 5 µL of the DNA sample was combined with 2 µL of loading dye (Safe super stain), and then applied to the gel wells for electrophoresis. The process consistently yielded high-quality, pure DNA samples. To identify the *ADORA2A* rs5751876 genotype of the participants, the amplification refractory mutation system-polymerase chain reaction (ARMS-PCR) technique was employed. For gene fragment amplification, a 20 µL master mix containing the Taq polymerase enzyme was prepared for each 0.2 mL microtube. The primers used included 1 µL of the forward primer for Allele C with the sequence 5′-TGAGCGGAGGCCCAATGGCAAC-3′, 1 µL of the forward primer for Allele T with the sequence 5′-TGAGCGGAGGCCCAATGGCAAT-3′, and a reverse primer with the sequence 5′-CTGGCACTGCTCTGTTACAACTCC-3′ (sourced from Macrogen, Seoul, Republic of Korea).

As previously mentioned, the PCR assays were conducted in a total volume of 25 μL comprising 3 μL of DNA, 1 μL of each primer, and 18 μL of PCR Master Mix from Thermo Fisher Scientific, Waltham, MA, USA. The thermocycling conditions involved an initial denaturation at 94 °C for 10 min, followed by 32 cycles of denaturation at 94 °C for 1 min, annealing at 65 °C for 1 min, and extension at 72 °C for 1 min, with a final elongation at 72 °C for 5 min (using a Thermal Cycler from Analytik Jena, Jena, Germany).

**Figure 2:**
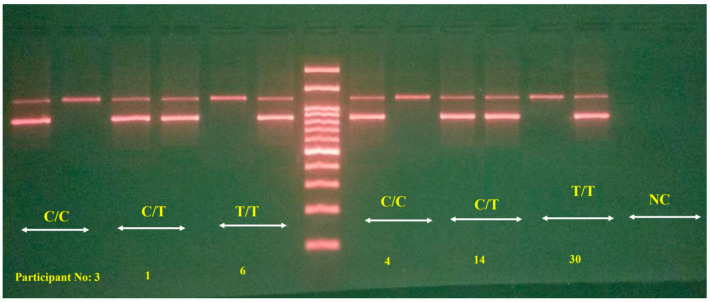
PCR products of *ADORA2A* gene amplification.

Post-PCR, the amplification products were visualized on a 2% agarose gel stained with ethidium bromide. Each sample underwent duplicate reactions to determine the presence of the C and T alleles. Identification of an 869-base pair (bp) band in both duplicate reactions signified a C/T heterozygous genotype, while detection of the 869-base pair band in either the C or T allele-specific reaction indicated homozygous C/C or T/T genotypes, respectively (Figure 2). An internal control involving primers for the mitochondrial genome, specifically the L strand primer 5′-CTCC ACCATTAGCACCCAAAGC-3′ and the H strand primer 5′-CCTA TTTGTTTATGGGGTGATG-3′, was included to generate a 250-base pair product, ensuring the validity of each reaction. All of the assays were performed in duplicate, alongside two negative controls.

#### 2.4.2. Cross-Sectional Study

Molecular genetic analysis was performed with DNA samples obtained from leukocytes (venous blood). Four milliliters of venous blood were collected in tubes containing EDTA (Vacuette EDTA tubes, Greiner Bio-One, Kremsmünster, Austria). DNA extraction and purification were performed using a commercial kit according to the manufacturer’s instructions (Technoclon, Moscow, Russia). HumanOmniExpressBeadChips (Illumina Inc., San Diego, CA, USA) or HumanOmni1-Quad BeadChips (Illumina, San Diego, CA, USA) were used to genotype the *ADORA2A* rs5751876 polymorphism, as previously described [35].

### 2.5. Biochemical Analysis

#### 2.5.1. Resistance Exercise Study

To measure the serum levels of GH and TS, 5 mL of blood was drawn from the anterior elbow vein of the subjects who had taken supplements and placebos pre-, post-, and 15 min after RE by the medical team at the gym. The blood samples for serum extraction were maintained at room temperature for 30 min on a rolling device to prevent clotting. Subsequently, the serum was separated using a centrifuge at 3000 rpm for 10 min. After transferring it into specialized tubes, the serum was frozen at −20 °C in the laboratory. The serum concentrations of GH (using the Monobind Inc. (Lake Forest, CA, USA) kit with a sensitivity of 0.104 international microunits/mL) and TS (using the IBL kit with a sensitivity of 0.07 ng/mL) were measured via the ELISA method.

#### 2.5.2. Cross-Sectional Study

Resting testosterone and growth hormone levels were examined in the serum of 94 athletes. A total of 10 mL of venous blood were collected the morning after an overnight fast and sleep in tubes containing EDTA and placed at 4 °C until processing (blood was collected at least 15 h after the last training). The testosterone and growth hormone were analyzed on a microplate spectrophotometer (Bio-Rad, Hercules, CA, USA) using an enzyme immunoassay test (Alkor-Bio, St Petersburg, Russia), as previously described [36].

### 2.6. Statistical Analysis

The data were analyzed using the Statistical Package for the Social Sciences (SPSS) v21.0 for Windows (IBM Crop, Armonk, NY, USA) or GraphPad InStat v3.05 (GraphPad Software, Inc., San Diego, CA, USA) software. Repeated measures analysis of variance and Bonferroni’s post hoc test were used to examine inter-group changes, while the independent *t*-test was employed to determine intra-group differences (analyzed with SPSS). Multiple regression was used to assess the relationship between the *ADORA2A* polymorphism and the resting levels of hormones in the athletes (adjusted for sex and age) (analyzed by GraphPad). A significance level of *p* ≤ 0.05 was utilized.

## 3. Results

### 3.1. Effect of ADORA2A Gene Polymorphism and Acute Caffeine Supplementation on Hormonal Response to Resistance Exercise

**Figure 3:**
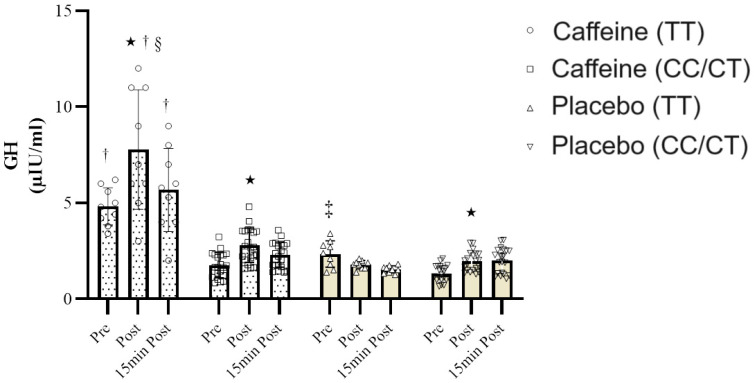
GH response to resistance exercise in subjects with *ADORA2A* TT and TC/CC genotypes in two conditions: caffeine consumption and placebo. Data presented as mean ± SD. * Significant differences with pre-resistance exercise. ^†^ Significant differences in carriers with CT/CC genotypes under CAF condition ^§^ Significant differences with PL condition. ‡ Significant differences in carriers with CT/CC genotypes under PL condition in pre-resistance exercise.

The analysis of the data on growth hormone (GH) levels revealed that Mauchly’s test of sphericity did not support the assumption of sphericity (*p* = 0.025). Consequently, the Greenhouse–Geisser correction was applied. The repeated measures analysis of variance demonstrated a notable time effect (F = 7.53, *p* = 0.001, ƞ2 =0.86), a significant time × condition interaction (F = 8.95, *p* < 0.001, ƞ2 = 0.91), and a substantial time × genotype × condition interaction (F = 5.81, *p* = 0.004, ƞ2 = 0.56). According to Bonferroni’s post hoc assessment, individuals with the TT genotype exhibited significantly heightened GH levels compared to those with the TC/CC genotypes in several instances: prior to testing (*p* = 0.012), immediately after testing (*p* = 0.007), and 15 min post-test (*p* = 0.001) under CAF consumption (Figure 3).

Under PL conditions, the only significant difference in GH levels between TT and TC/CC genotypes was detected prior to testing (*p* = 0.035). Furthermore, post-test GH concentrations in the TT genotype were significantly elevated compared to the placebo (*p* = 0.05), yet no such difference was observed for the TC/CC genotype between the CAF and PL conditions. Additionally, an independent samples *t*-test with Bonferroni correction was used to investigate differences within the groups concerning GH concentration. The findings indicate a significant rise in post-test GH levels in the TT genotype under CAF conditions compared to pre-test levels (*p* ≤ 0.05). However, for individuals carrying the TC/CC genotypes, significant increases in GH were noted both post-test and 15 min post-test in comparison to pre-test levels when both CAF and PL were consumed (*p* ≤ 0.05).

**Figure 4:**
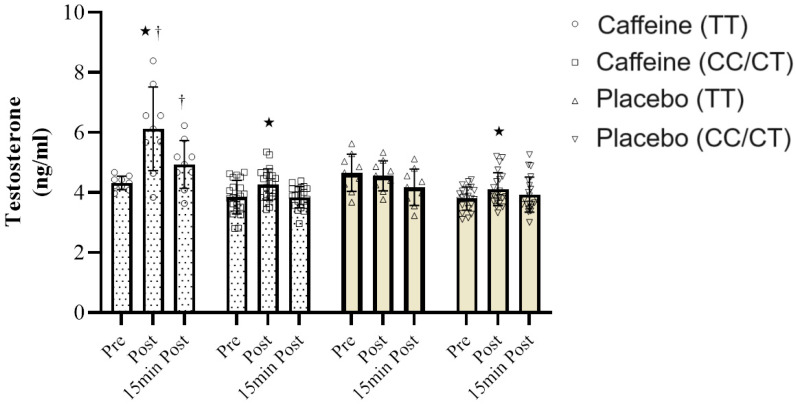
TS response to resistance exercise in subjects with *ADORA2A* TT and TC/CC genotypes in two conditions: caffeine consumption and placebo. Data presented as mean ± SD. * Significant difference with pre-resistance exercise. ^†^ Significant difference in *ADORA2A* CT/CC individuals under CAF condition.

Regarding TS, the outcomes of Mauchly’s test of sphericity indicate that the assumption of sphericity was acceptable concerning TS (*p* = 0.42). The results of the repeated measures analysis of variance demonstrate significant disparities in the effect of time (F = 18.39, *p* = 0.0001, ƞ2 =0.66), time × condition interaction (F = 10.65, *p* = 0.0001, ƞ2 = 0.72), and time × genotype × condition interaction (F = 8.42, *p* = 0.0001, ƞ2 = 0.58) (Figure 4). Hence, it can be concluded that pre-resistance exercise CAF supplementation significantly affects TS concentration, based on the *ADORA2A* genotype.

To assess differences between the groups, Bonferroni’s post hoc test results revealed that under CAF consumption, the TS concentration was notably higher in the TT genotype compared to carriers of the TC/CC genotypes post-RE (*p* = 0.001) and 15 min post-RE (*p* = 0.026). Additionally, an independent samples *t*-test with Bonferroni correction was employed to examine intra-group differences related to the TS concentration. These findings indicate that a significant increase in TS was observed post-RE compared to pre-RE, specifically in the condition of CAF consumption in the TT genotype (*p* ≤ 0.05). Conversely, for TC/CC genotype carriers, a significant increase in TS concentration was noted post-RE compared to pre-RE, both for CAF consumption and PL (*p* ≤ 0.05).

### 3.2. Association between ADORA2A Genotypes and Resting Hormone Levels in Athletes

In the group of athletes, carriers of the *ADORA2A* TT genotype (*n* = 12) had significantly higher testosterone levels (males: 24.9 (3.4) vs. 22.1 (6.6) nmol/L; females: 2.0 (0.6) vs. 1.7 (0.7) nmol/L; *p* = 0.0125 adjusted for age and sex) compared to the CT and CC genotypes carriers (*n* = 82). Furthermore, carriers of the *ADORA2A* TT genotype had significantly higher growth hormone levels (males: 3.0 (4.9) vs. 1.9 (3.9) ng/mL; females: 9.6 (4.8) vs. 5.5 (6.0) ng/mL; *p* = 0.0365 adjusted for age and sex) compared to the CT and CC genotype carriers.

## 4. Discussion

There is growing evidence that various genetic polymorphisms may underlie individual responses to dietary supplements, including caffeine and whey protein [32,37,38]. In the current study, we demonstrated that the *ADORA2A* gene rs5751876 polymorphism may modify the effect of caffeine intake on the hormonal response to resistance exercise. Although the designs of our two studies (resistance exercise and cross-sectional) are different, we believe that the cross-sectional study may represent the chronic effect of caffeine intake on TS and GH levels in athletes who were light caffeine consumers, thus partly replicating findings from the resistance exercise study.

Prior research has illustrated that caffeine exhibits anti-inflammatory [30,39], metabolic [40], anti-apoptotic [41], lipolytic [26], and sports performance-enhancing properties [42]. Although few studies address the impact of caffeine supplementation on GH and TS levels, the existing results are conflicting [22,24,26]. This study uniquely demonstrates that the consumption of 6 mg/kg of caffeine supplemented one hour before resistance activity leads to a significant increase in the serum concentration of anabolic hormones (GH and TS) in male athletes homozygous for the *ADORA2A* T allele after resistance activity.

In terms of the ergogenic effect of caffeine, previous studies have generally indicated a performance-enhancing effect [43,44], although contradictory results have been reported in some cases [44,45]. This inter-individual variation in response to caffeine consumption may stem from genetic variations in genes responsible for caffeine metabolism or sensitivity, such as *CYP1A2* and *ADORA2A* [29,44]. Previous research has revealed that the *ADORA2A* genotype may influence the effect of caffeine on sports performance improvement. Loy et al. [29] demonstrated for the first time that untrained women with the *ADORA2A* rs5751876 TT genotype, who consumed 5 mg of caffeine per kg of body weight 60 min before cycling, exhibited greater work output during a subsequent time trial compared to C allele carriers.

Conversely, Grgic et al. [43] showed that resistance-trained subjects with the *ADORA2A* rs5751876 C allele experienced caffeine ergogenic effects similar to the TT genotype after caffeine consumption, and Carswell et al. [28] reported that both TT and C allele carriers displayed similar improvements in exercise performance during a 15-minute time trial after caffeine consumption.

Caffeine acts as a competitive adenosine antagonist at the A1, A2A, A2B, and A3 receptors, with antagonistic effects believed to underlie many of its physiological impacts [3]. Among these receptors, caffeine exhibits the highest affinity for A1 and A2A receptors. Adenosine A1 and A2A receptors are expressed in many tissues, including the brain, skeletal muscle, kidney, spinal cord, adipose tissue, and immune cells such as neutrophils. A2A receptors are involved in motor control and stimulation of action because A2ARs are widely expressed on striatum neurons affecting the basal ganglia, and contribute to motion control and motivation [46]. According to the GTEx portal, the rs5751876 variation significantly affects *ADORA2A* gene expression in various tissues, including the basal ganglia, testis, prostate, and lung [47]. Caffeine can stimulate A2A receptors, which may be modified by genetic variation, enhancing dopamine transmission, and thus influencing motor activity [1,48]. 

Variances in caffeine consumption and response have been observed among individuals with genetic variations in the *ADORA2A* gene (rs5751876) [49]. Given that homozygous T allele (TT) individuals have higher testosterone and growth hormone levels (according to our study), it is possible that TT genotype carriers experience greater improvements in exercise performance with caffeine consumption [29].

Interestingly, *ADORA2A* gene expression is positively associated (*p* = 0.0002, adjusted for age and sex) with androgen receptor (*AR*; mediates action of testosterone) gene expression in m. gastrocnemius in the GTEx cohort [47], which may partly explain and support our observation. Currently, no research has explored the effects of caffeine consumption on circulating GH and TS following resistance exercise concerning *ADORA2A* gene variants. However, conflicting findings have been reported regarding the effects of caffeine on GH and TS levels during resistance activity [22,24,50].

In one study, the impact of 6 mg/kg caffeine was investigated using a counterbalance method on TS, GH, insulin, cortisol, free fatty acids, and lactate levels after 3 sets with 10 repetitions at 75% 1RM in resistance training subjects. The results indicated a significantly higher concentration of free fatty acids in the caffeine condition than in the control condition [26]. However, the GH response to caffeine was notably lower than in the control condition. No significant differences were observed in other variables between the two conditions. Additionally, a decrease in GH secretion following the consumption of a caffeinated beverage after resistance activity has been documented [50]. Conversely, it has been reported that there was a significant increase in GH concentration immediately after and 15 min after resistance activity in trained men, with resistance observed in the caffeine consumption condition compared to the placebo [44].

Godfrey et al. [51] indicated that growth hormone secretion significantly increases after subjects engage in resistance exercises with moderate intensity and high repetitions, a finding that was primarily attributed to the rise in nitric oxide and lactate. Nitric oxide serves as a crucial intracellular and intercellular transmitter, playing a pivotal role in regulating the release of growth hormone from the hypothalamus–pituitary axis. As a result, nitric oxide appears to facilitate the release of growth hormone from the anterior pituitary into the bloodstream.

Caffeine’s impact on sports performance is governed by three primary mechanisms: elevation of cAMP leading to increased lipolysis, mobilization of intracellular calcium from the sarcoplasmic reticulum, and acting as a competitive antagonist of adenosine receptors in the central nervous system [52]. This action can notably enhance performance, particularly during endurance activities. The augmentation of calcium release from the sarcoplasmic reticulum is also suggested as a mechanism that contributes to the ergogenic effect of caffeine on sports performance. Considering the dosage utilized in the current study, it appears unlikely that this mechanism would be operational, as it typically occurs at levels higher than the physiological 6 mg/kg values [52].

In this study, the consumption of 6 mg of caffeine per kilogram of body weight one hour before resistance activity resulted in increased GH and TS levels in athletes. Furthermore, the effect of caffeine on the concentration of these hormones was more pronounced in athletes with the TT genotype than in those with TC/CC genotypes. These results were partly validated in the cross-sectional study, with TT homozygotes showing higher levels of TS and GH compared to C allele carriers in the resting state. Consequently, it appears that athletes with the TT genotype are likely to experience more pronounced anabolic effects following resistance activity with caffeine consumption. Evidence suggests that higher testosterone levels may provide an athletic advantage [53,54,55,56]. Therefore, we speculate that carrying the *ADORA2A* rs5751876 TT genotype may be beneficial for power performance.

There are some limitations that should be acknowledged. Firstly, the size of the two independent cohorts was relatively small (resistance exercise study) to moderate (cross-sectional study). Thus, extension and replication within groups of differing geographic ancestry are needed to translate these findings more broadly. Secondly, functional studies are necessary to establish a causal relationship between the *ADORA2A* genotype and hormonal responses to exercise following caffeine supplementation.

## 5. Conclusions

In conclusion, the *ADORA2A* rs5751876 TT genotype is associated with pronounced anabolic effects following resistance activity and caffeine consumption, which may partly explain better ergogenic effects of caffeine intake in TT homozygotes in response to exercise. Our findings support the idea that inter-individual differences in sport and exercise performance following caffeine ingestion may be attributed to genetic variations associated with caffeine metabolism or sensitivity [57].

## Data Availability

The data that support the findings of this study are available from the corresponding author upon reasonable request due to privacy of athletes.
